# Supplementary material for: Chromothripsis during telomere crisis is independent of NHEJ, and consistent with a replicative origin
Source: Genome Res. 2019 May;29(5):737–49. doi: 10.1101/gr.240705.118 (PMC6499312; doi:10.1101/gr.240705.118)
Supplement: Supplemental Material [file supp_gr.240705.118_Supplemental_file_1.zip › contigs/annotated_contigs/DB96/contig.2.DB96_length_578_mean_cov_7.94809688581.docx]

**DB96_length_578_mean_cov_7.94809688581**

```
TCATCAGCAAATCCAGAAAACAACCTTTCCTGGAACTTTCTGACCACAGAAATCTCATCCCAGATCTGAAACTTAAGCAGAAGGCAAGA
 >chr11:14814371-14814731 - E=5e-202
TTAAGGGTCATAATCTCATATGGATACCATACCTCTGTAGGACACTTGTTAAGCAGCCATTTGTCTTCAAAGATTCATTTTAATGCAAT

CTCGAACTCTTTACAGAACTTAAACAGGCTTTAGAGGAGTAGTAATGGCTGACATAAAGTCAAATGTGAAAAGCGCCGTAAGAGAATGT

GTTATGGAAATTCAGAGGAAGAAAAGAGTACTTCTCCTTGGAGAAGGGAGAAGGACCTCCAGGTGTTATCCTTATAAGGTGATTTATGA

GCCC|TTCTTTGCCTTTTTTGGTTTAAAGTCTGTTTTGTCTGAAATTAGGATTGCAACCTCTGCTTTTTCCTGATTTCCACTTGCTTGG
 >chr11:14846862-14847094 - E=3e-109
TAGATTTTCCTCCATCCCTTTGTTTTGAGCCTATGGGTGTTGCTGCCTGTGAAATGGGTCTCTTGAAGACAGTGTATCATTGGGTCTTA

CTTTTTTTAAAATCCAGTTTGCCACTCTATGCCTCTTAAATGGGG
```
